# Identification of equine herpesvirus 8 in donkey abortion: a case report

**DOI:** 10.1186/s12985-021-01738-2

**Published:** 2022-01-06

**Authors:** Tongtong Wang, Leyu Hu, Yonghui Wang, Wenqiang Liu, Guiqin Liu, Mingxia Zhu, Wei Zhang, Changfa Wang, Huiying Ren, Liangliang Li

**Affiliations:** 1 Research Institute of Donkey High-Efficiency Breeding and Ecological Feeding, College of Agronomy, Liaocheng University, Liaocheng, China; 2 College of Veterinary Medicine, Qingdao Agricultural University, Qingdao, China; 3 Dairy Cattle Research Center, Shandong Academy of Agricultural Sciences, Ji’nan, 250100 China

**Keywords:** Donkey, Abortion, Equid herpesviruses 8, Virus isolation

## Abstract

**Background:**

Equine herpesvirus-8 (EHV-8) is one of the most economically significant viruses that infect mammals of the genus Equus worldwide, which cause severe respiratory diseases and abortion in horses. However, there is no report of abortion caused by EHV-8 in donkeys.

**Case presentation:**

The present case report is about a 4-year-old donkey having an abortion and showing a serious respiratory issue on the 296th day of pregnancy. Bacteriological and molecular tests were used to screen possible bacterial/viral pathogens to detect the etiological agent. *Salmonella abortus equi*, EHV-1, EHV-4, and EAV were all negative in the current study. EHV-8, on the other hand, was the only agent that was isolated and identified.

**Conclusions:**

This was for the first time that EHV-8 had been isolated from a donkey in China. EHV-8 infection can cause abortion in donkeys; therefore, veterinarians and breeders should be aware of it.

**Supplementary Information:**

The online version contains supplementary material available at 10.1186/s12985-021-01738-2.

## Background

The primary infectious pathogens affecting the horse or donkey industries are equine herpesviruses (EHVs) [1]. Nine herpesviruses have been found in equids so far. EHV-1, EHV-2, EHV-3, EHV-4, and EHV-5 infect the horse, while EHV-6 (asinine herpesvirus, AHV-1), EHV-7 (AHV-2), and EHV-8 (AHV-3) are associated with infections in donkeys [2–4]. AHVs, EHV-8, and EHV-9, in particular, are more closely linked to EHV-1 than EHV-4 [5, 6].

EHV-8 is a Herpesviridae virus with a double-stranded enveloped DNA that belongs to the alphaherpesvirinae subfamily [4, 7]. EHV-8's genome is around 150 kb long and contains 76 open reading frames (ORFs) [8]. In 1988, EHV-8 was isolated in the nasal cavity of latently infected donkeys in Australia [3]. It was also identified from a horse with fever and nasal discharge in China and donkeys in Israel with severe post-castration incisional infection [9, 10]. EHV-8's pathogenesis is unknown, and the virus has only been linked to respiratory and neurological illness in donkeys, as well as miscarriage in horses [4, 5].

Here is the first report documenting a typical case of EHV-8-induced abortion in a donkey. It has been proposed that EHV-8 is a potential pathogen linked to donkey abortion.

## Case presentation

**Fig. 1:**
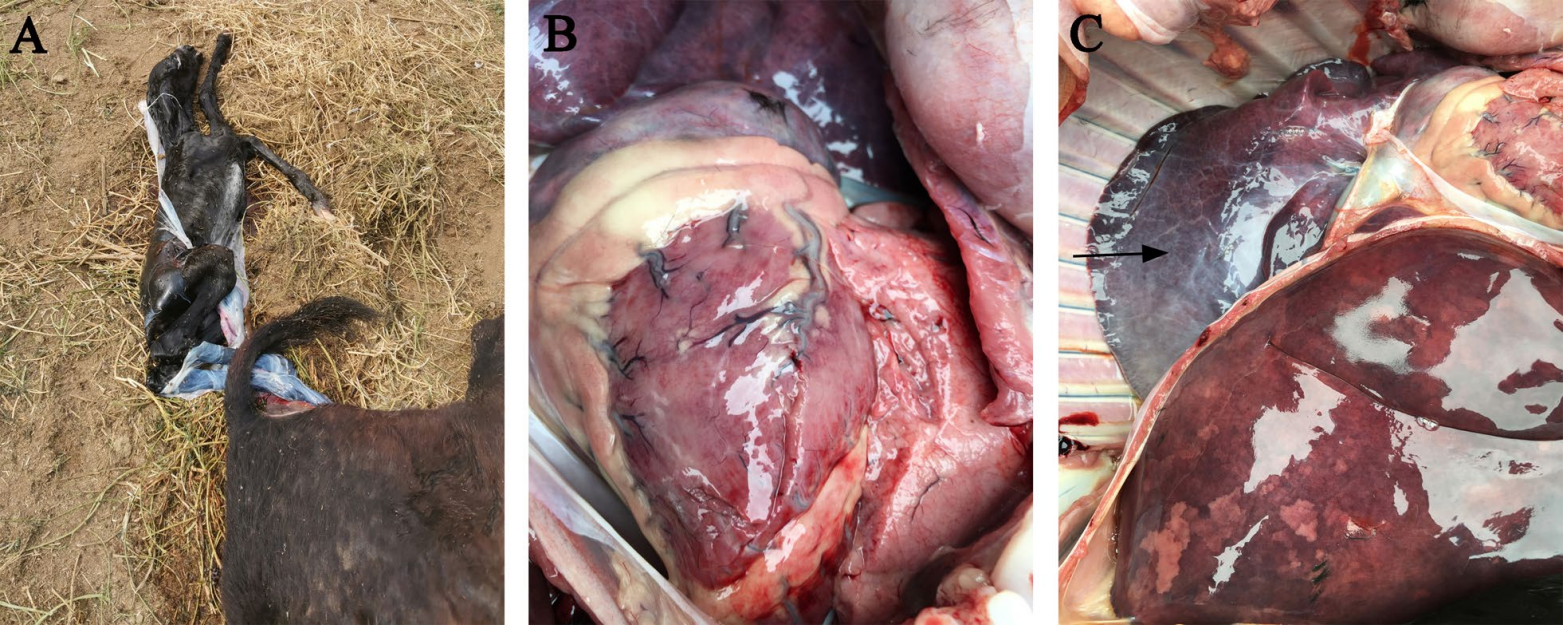
Gross lesions of an aborted fetus. **a** Aborted fetuses of donkey; **b** Heart; **c** Lungs and liver

A 4-year-old female donkey from a large-scale farm in Liaocheng, China, had an abortion on the 296th day of pregnancy on May 29, 2021. Anorexia, sadness, unwillingness to move, and a bent head were all symptoms of the female donkey. Except for the lungs, which had a blue/purple tinge, necropsy of the aborted fetus revealed no visible gross abnormalities (Fig. 1).

**Fig. 2:**
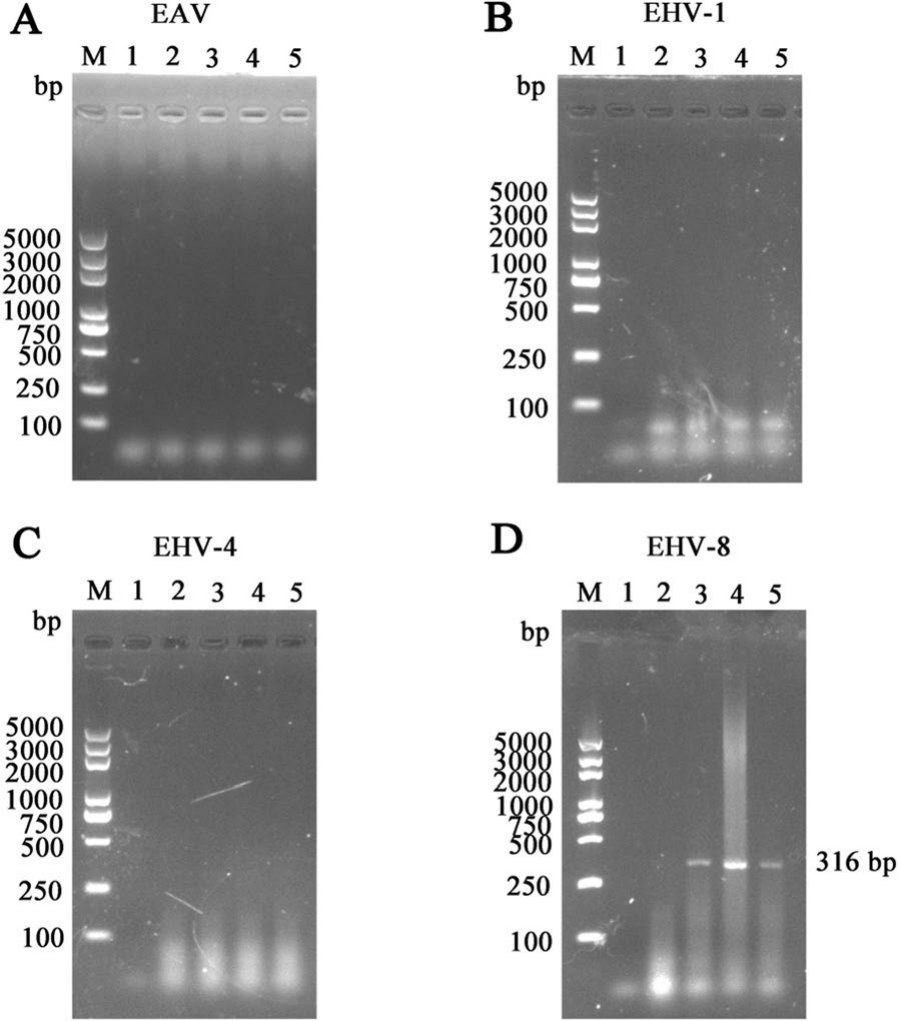
Screening of virus pathogens. Viral DNA/RNA was extracted from tissues, and EAV (**a**), EHV-1 (**b**), EHV-4 (**c**), EHV-8 (**d**) were detected by RT/PCR and PCR. Lane M represents a 5000 bp DNA molecular weight ladder. 1 represents negative control, 2 represents liver, 3 represents placenta, 4 represents umbilical cord, 5 represent lungs

The tissue specimens of the placenta, umbilical cord, lungs, and liver from the aborted fetus were taken for bacteriological testing to identify the etiological agent. As previously described, all tissue specimens were cultivated on Salmonella-Shigella agar and 5% sheep blood agar and incubated at 37 °C for 24 h [11]. There were no bacterial colonies on the culture plates, according to the results. Following that, as stated in earlier publications, potential viruses were identified [12]. An EasyPure® Viral DNA/RNA Kit was used to extract RNA and DNA from the placenta, umbilical cord, lungs, and liver tissues (Beijing TransGen Biotech Co., Ltd., China), according to the manufacturer's instructions. A PrimeScript™ RT-PCR kit was used to perform One-Step reverse transcription-polymerase chain reaction (RT-PCR) amplification using appropriate primers for EAV detection (Takara Bio, Inc., Japan). PCR was used to test for EHV-1, EHV-4, and EHV-8 using specified primers (Table 1). The PCR cycle was as follows: 94 °C for 5 min, followed by 30 cycles at 94 °C for 30 s, 56 °C for 45 s, 72 °C for 1 min and with a final extension at 72 °C for 10 min. EAV, EHV-1, and EHV-4 were all found to be negative in all of the tissue samples. The umbilical cord, placenta, and lung tissues were all EHV-8-positive with a predicted 316 bp product. However, no detection in the liver tissues and identified using an agarose gel (Fig. 2).

**Table 1:** The primer sequences in this study

Primers	Primer sequences (5'–3')	PCR product sizes (bp)
EAV ORF7-F	ATGGCGTCAAGACGATCAC	333
EAV ORF7-R	TTACGGCCCTGCTGGAGGC	
EHV-1 gB-F	GAACCTCAGCCAACCCA	792
EHV-1gB-R	GCACTTTGCGGACGAAC	
EHV-4 gB-F	CTTAATCGCATTTAGACCGATG	1591
EHV-4 gB-R	CCGGAACTAGAAAGATGTTATGC	
EHV-8 G1-F	TCAGACTGTCACTCGTGGGA	316
EHV-8 G1-R	CCTGGAGGCCGTTTAACACA

**Fig. 3:**
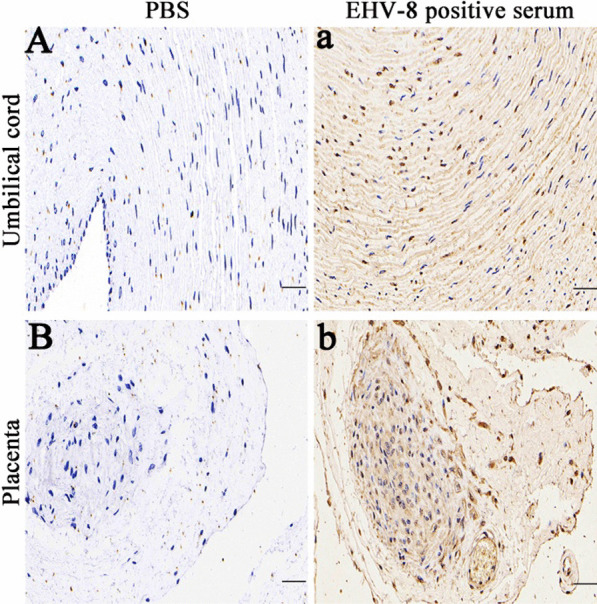
Representative images of immunohistochemistry (IHC) staining for EHV-8 using the positive serum. IHC was performed to detect the EHV-8 antigen. The PBS-treated group was negative control, umbilical cord (**A**) and placenta (**B**). The experimental group was treated with EHV-8 positive serum on the umbilical cord (**a**) and placenta (**b**) tissues. Scale bars, 50 μm

Immunohistochemistry (IHC) was used on the placenta and umbilical cord to confirm EHV-8 infection in vivo, as described before [13–15]. Briefly, The umbilical cord and placenta tissue were preserved in 10% formalin, embedded in paraffin wax, cut to 5 μm using a microtome (Leica), and mounted on slides. The slides were treated with hydrogen peroxide to inhibit endogenous peroxidase. After overnight immunostaining with EHV-8-positive serum (our lab stocks), slides were rinsed in PBS, treated with horseradish peroxidase-conjugated rabbit anti-donkey IgG for 1 h, stained with diaminobenzidine for 5 min, rinsed in water, counterstained in Gill's hematoxylin for 30 s, dehydrated, cleared, and placed on a coverslip. Negative controls were treated identically to positive controls without the antibody incubation to evaluate non-specific binding effects. Because of the presence of diaminobenzidine, EHV-8-positive cells in the umbilical cord and placenta looked brown, as shown in Fig. 3, but no EHV-8-positive cells were seen in the control slides.

**Fig. 4:**
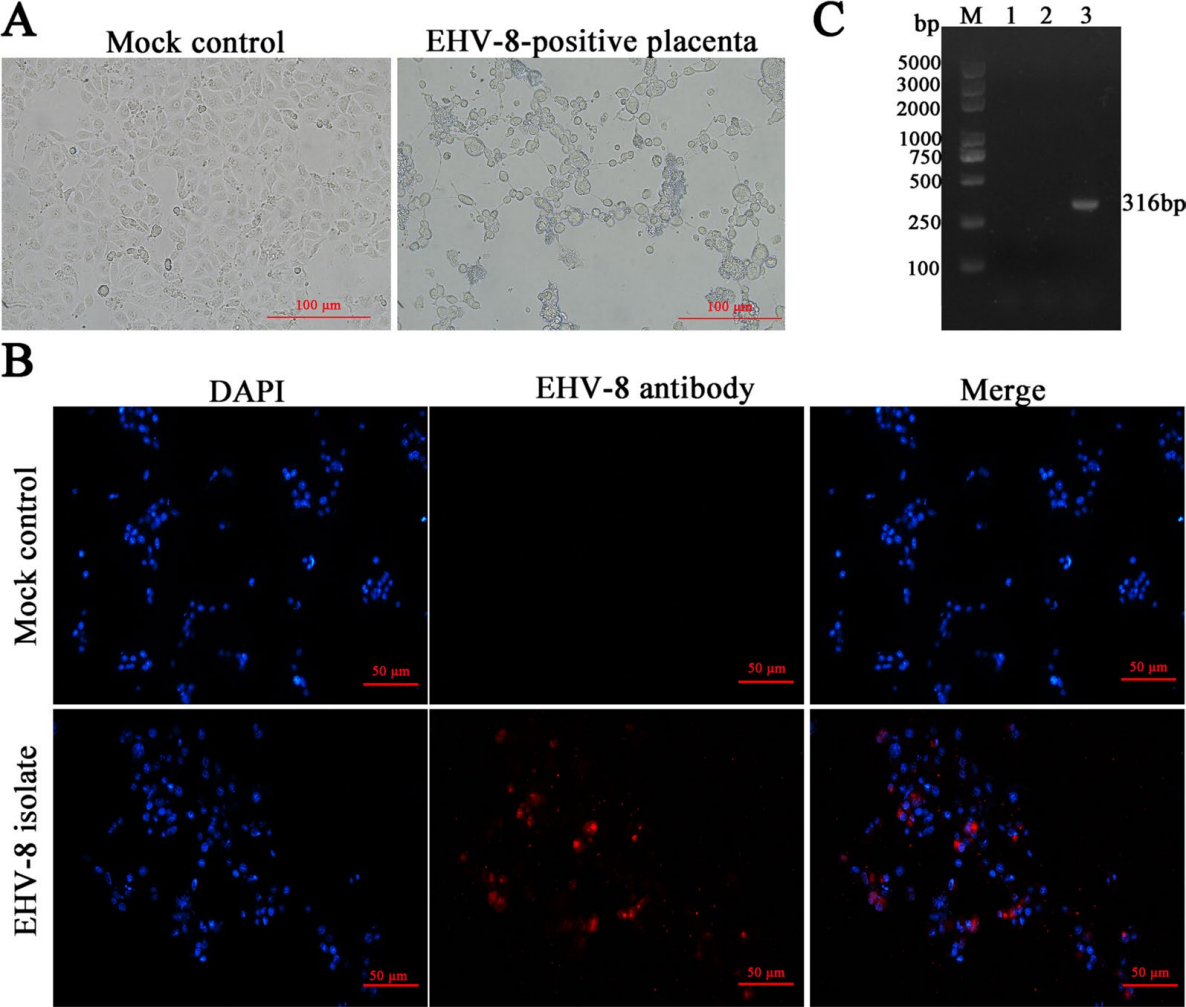
Isolation and identification of EHV-8. The RK-13 cells were inoculated with supernatant of EHV-8-positive placenta (right panel) or mock control (left panel). **a** A total of 48 h post-infection, the CPE was observed using microscopy. Scale bars, 100 µm. **b** Identification of EHV-8 isolate by IFA. CPE-positive RK-13 cells and mock control cells were fixed with 75% alcohol. Images represent the subcellular locations of EHV-8 proteins using indirect immunofluorescence detection using anti-EHV-8 mouse serum, and the corresponding DyLight 594-conjugated secondary antibodies. Cells were imaged by Leica DMi8. Scale bars, 50 µm. **c** PCR detection of the EHV-8 ORF70 genes from a different group. The DNA was extracted from these cells. PCR products were electrophoresed in a 1% agarose gel. Marker (lane M) was included on the left, 1 represents negative control, 2 represents mock control RK-13 cell, 3 represents the CPE positive RK-13 cells

Following that, the EHV-8 strain was isolated as described previously [16]. The cytopathic effect (CPE) was seen in RK-13 cells (right) and negative cells (left) after two days of inoculation with the supernatant from an EHV-8-positive placenta (Fig. 4a). In order to evaluate EHV-8 isolate, the immunofluorescence assay (IFA) were performed with anti-EHV-8 mouse serum (made in our lab), the CPE-positve cells and mock cells were fixed with 75% alcohol repectively, a DyLight 594-labelled Goat Anti-Mouse IgG(H + L) served as second antibody. Comparing with mock control RK-13 cells, EHV-8 proteins were observed in the cytoplasm and nucleus in CPE-positive cells (Fig. 4b).

Further, the EHV-8 isolate was then tested using PCR, the CPE-positive cells were collected, and DNA was extracted for EHV-8 detection using G1-Forward and G1-Reverse primers. In the CPE-positive group, an agarose gel electrophoresis revealed a single band with an anticipated size of 316 bp (Fig. 4c).

Meanwhile, after a week of antibiotic treatment, the female donkey gradually recovered. The female donkey's nose swabs and blood samples were obtained and utilized to test for EHV-8 infection using PCR as described above. However, the *ORF70* gene's incomplete sequence is still detectable in these samples (Additional file 1: Figure S1). Finally, the donkey was put down by the owner to prevent EHV-8 from spreading.

## Discussion and conclusions

A slew of large-scale donkey farms has sprung around China in recent years, contributing considerably to the local economy. However, pandemic illnesses have grown year after year due to the extensive breeding of donkeys [17–19]. Abortions and respiratory illnesses, in particular, have caused significant economic losses for the donkey business in China, severely limiting its expansion. According to previous studies, abortion in donkeys or equids is associated with several microbes, such as *Salmonella abortus equ*, *Leptospira spp*, *Streptococcus equi ssp, Zooepidemicus*, equine arteritis virus (EAV), EHV-1, EHV-4, and EHV-8 [4, 14, 20–25]. In a previous study by Li et al., *Salmonella abortus equi* was reported closely linked with female donkey abortions, although not found in this instance [12]. Furthermore, EHV-1, EHV-4 and EAV were negative in this instance, as in the previous study [12].

EHV-8 has been linked to respiratory symptoms, miscarriages, and neurologic illness in donkeys and horses [9, 10]. Bacteriology culture and viral screening were conducted in this instance. Only EHV-8 was discovered and isolated, suggesting that EHV-8 is one of the primary pathogenic agents that cause abortion in female donkeys.

Based on our recent study, the positive rate of EHV-8 from large-scale donkey farms in Shandong province might be as high as 25.3 percent. As a result, EHV-8 infection in donkey farms should be given more attention, and suitable preventative measures should be explored to reduce EHV-8 infection.

## Supplementary Information

**Additional file 1:** EHV-8 infection detected from female donkey by PCR.

## Data Availability

All relevant data are within this paper.

## Abbreviations

EHV-8: Equine herpesvirus-8
EAV: Equine arteritis virus
ORFs: Open reading frames
CPE: Cytopathic effect
