# Supplementary material for: Identification of equine herpesvirus 8 in donkey abortion: a case report
Source: Virol J. 2022 Jan 6;19:10. doi: 10.1186/s12985-021-01738-2 (PMC8734136; doi:10.1186/s12985-021-01738-2)
Supplement: Supplementary file 1 — Additional file 1: EHV-8 infection detected from female donkey by PCR. [file 12985_2021_1738_MOESM1_ESM.docx]

**Figure** [**legend**](javascript:;)


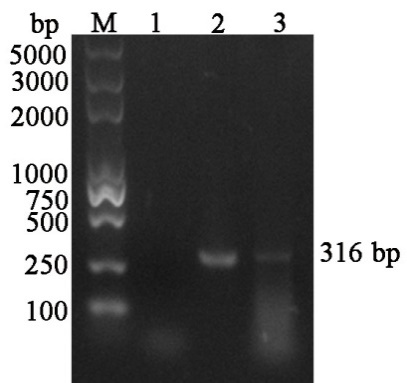


**Figure S1**. **EHV-8 infection detected from female donkey by PCR.** Viral DNA was extracted from nasal swabs and blood sample. PCR products were electrophoresed in a 1% agarose gel. Marker (lane M) was included on the left, 1 represents negative control, 2 represent blood, 3 represent nasal swabs.
